# MPT0G413, A Novel HDAC6-Selective Inhibitor, and Bortezomib Synergistically Exert Anti-tumor Activity in Multiple Myeloma Cells

**DOI:** 10.3389/fonc.2019.00249

**Published:** 2019-04-09

**Authors:** Fang-I Huang, Yi-Wen Wu, Ting-Yi Sung, Jing-Ping Liou, Mei-Hsiang Lin, Shiow-Lin Pan, Chia-Ron Yang

**Affiliations:** ^1^School of Pharmacy, College of Medicine, National Taiwan University, Taipei, Taiwan; ^2^Ph.D. Program for Cancer Molecular Biology and Drug Discovery, College of Medical Science and Technology, Taipei Medical University and Academia Sinica, Taipei, Taiwan; ^3^Ph.D. Program in Biotechnology Research and Development, College of Pharmacy, Taipei Medical University, Taipei, Taiwan; ^4^School of Pharmacy, College of Pharmacy, Taipei Medical University, Taipei, Taiwan; ^5^Graduate Institute of Cancer Molecular Biology and Drug Discovery, College of Medical Science and Technology, Taipei Medical University, Taipei, Taiwan; ^6^Biomedical Commercialization Center, Taipei Medical University, Taipei, Taiwan

**Keywords:** multiple myeloma cells, histone deacetylase 6, bortezomib, combination therapy, bone marrow stromal cells, synergistic effect

## Abstract

In multiple myeloma (MM), homeostasis is largely maintained by misfolded protein clearance via the proteasomal and aggresomal pathways. Histone deacetylase 6 (HDAC6) binds polyubiquitinated proteins and dynein motors and transports this protein cargo to the aggresome for further degradation. Accordingly, a combination of an HDAC6 inhibitor and bortezomib (BTZ) could increase ubiquitinated protein accumulation, leading to further apoptosis. Here we evaluated the anti-MM activity of MPT0G413, a novel specific HDAC6 inhibitor, using *in vitro* and *in vivo* models. MPT0G413 treatment more significantly inhibited cell growth in MM cells than in normal bone marrow cells. Furthermore, the combination of MPT0G413 and BTZ enhanced polyubiquitinated protein accumulation and synergistically reduced MM viability, increased caspase-3, caspase-8, caspase-9 levels, and cleaved poly (ADP) ribosome polymerase and also inhibited adherence of MM cells to bone marrow stromal cells (BMSC) and reduced VEGF and IL-6 levels and cell growth in a co-culture system. The combination treatment disturbed the bone marrow microenvironment and induced synergic, caspase-dependent apoptosis. Xenograft tumor growth significantly decreased in combination-treated SCID mice. In conclusion, MPT0G413 and BTZ synergistically inhibit MM viability, providing a framework for the clinical evaluation of combined therapies for MM.

## Introduction

Multiple myeloma (MM) is a B cell malignancy characterized by the proliferation of bone marrow (BM) plasma cells and the production of large amounts of abnormal immunoglobulins (1) In the United States, it was estimated that 30,770 new MM cases would be diagnosed in 2018, accounting for 1.8% of newly diagnosed cancer cases (2). Furthermore, 12,770 MM-related deaths in 2018 accounted for an estimated 2.1% of all cancer deaths (2).

In the past decade, MM treatment outcomes have improved since the approval of thalidomide-related immunomodulatory drugs and the proteasome inhibitor bortezomib by the FDA. Although MM patients have exhibited good responses to these agents (3), the incidence of relapse remains high (4, 5). Therefore, new agents are needed to ensure better long-term outcomes for such patients.

Histone deacetylases (HDACs) are enzymes that remove acetyl groups from lysines of histones and thus, act as gene transcription regulators (6). HDACs are considered attractive cancer therapeutic targets because changes in histone modification are frequently observed in human cancers, including MM. Accordingly, four small-molecule pan-HDAC inhibitors have been introduced for hematologic malignancies (7, 8). Recent clinical studies have evaluated the use of pan-HDAC inhibitors, such as vorinostat (suberoylanilide hydroxamic acid, SAHA) or panobinostat (LBH589) in combination with bortezomib (BTZ) to inhibit both proteasomal and aggresomal protein degradation and overcome clinical resistance to BTZ (9). However, the side effects of regimens combining pan-HDAC inhibitors and BTZ, which include fatigue, diarrhea, nausea (10), QT-interval prolongation (11, 12), and thrombocytopenia (13), limit the clinical utility of these combination treatments (14).

HDAC6, a member of the class IIB HDAC family, contains two catalytic domains and a C-terminal zinc finger domain that binds free ubiquitin as well as mono- and polyubiquitinated proteins with high affinity (15). This unique cytoplasmic deacetylase can deacetylate substrates such as tubulin, heat shock protein 90 (HSP90), and cortactin (16–18). A previous report has demonstrated that HDAC6-deficient mice are viable and normally develop, indicating that HDAC6 inhibition would not cause severe side effects (19). Furthermore, HDAC6 plays an important role in misfolded/unfolded protein degradation, in addition to its roles in cell morphology, adhesion, migration, and tumor cell invasion/metastasis (20).

For the production of high amount of abnormal immunoglobulins, MM cells heavily depend on misfolded/unfolded protein clearance mechanisms, particularly the proteasomal and aggresomal pathways, to maintain homeostasis (21, 22). Proteasomes are abundant multi-enzyme complexes that provide the main pathway for degradation of intracellular protein and thus, help in the removal of misfolded/unfolded proteins (21). The FDA has approved bortezomib, a proteasome inhibitor that blocks the proteasomal degradation of abnormal protein. Although this drug prolongs survival in MM patients, its long-term treatment has been shown to lead to drug-resistant relapse in most patients (23), as mentioned previously. In addition, proteasome inhibition has multifactorial downstream biological effects and directly affects both MM cells and the BM microenvironment via the inhibition of cytokine secretion, suppression of adhesion molecule expression, and inhibition of angiogenesis (22). The aggresomal pathway degrades ubiquitinated misfolded/unfolded proteins and ultimately induces the autophagic clearance of these proteins via lysosomal degradation (24). HDAC6 plays an important role in this pathway because it can bind both polyubiquitinated proteins and dynein motors, thus recruiting the protein cargo to dynein motors for further autosomal degradation (24).

A recent study showed that ACY-1215, a HDAC6 inhibitor currently in phase II clinical trials, could suppress the growth of MM when administered as a component of combination therapy (25). Therefore, a combination of an HDAC6 inhibitor and BTZ could increase the accumulation of ubiquitinated proteins and enhance BTZ-induced cell cytotoxicity. Several studies also indicated combination HDAC6 inhibitors with anticancer agents provide strong scientific rationale in the clinical setting of hematological malignancies (26). However, the mechanism by which this combination therapy would affect the interactions of MM cells with bone marrow stromal cells (BMSCs) remains unclear.

We previously developed a series of 5-aroylindolyl-substitued hydroxamic acids compounds with potential HDAC6 inhibitory activity. Among them, N-hydroxy-4-((5-(4-methoxybenzoyl)-1H-indol-1-yl)methyl)benzamide (MPT0G413) exhibited potent and selective inhibitory activity against HDAC6, with an IC_50_ value of 3.92 nM and 100-fold greater selectivity for HDAC6 relative to other HDACs isoforms (27). However, the molecular action by which MPT0G413 inhibits the growth of MM cells has not been clearly elucidated. In this study, we evaluated the anticancer activity of a combination of the specific HDAC6 inhibitor MPT0G413 and BTZ in both *in vitro* and *in vivo* models and studied the effects of this combination therapy on parameters such as cytokine secretion and cell adhesion in a microenvironment comprising MM cells and BM. Our results demonstrate that the combination of MPT0G413 and BTZ not only induced synergic apoptosis in MM cells, but also downregulated VEGF, IL-6 secretion to inhibit MM growth in a MM/BMSC co-culture system. From a translational perspective, these findings could potentially improve the efficacy of anti-MM treatment.

## Materials and Methods

### Materials

MPT0G413 were synthesized by Professor Jing-Ping Liou, and the purities were > 98%. We used non-conjugated primary antibodies against HDAC6 (#7612), Caspases-3 (#9661),−8 (#9746), and−9 (#9502), acetyl-histone 3 (#9677), acetyl-histone 4 (#8647), histone 3 (#9715), histone 4 (#2935), acetyl-α-tubulin (#5335), were purchased from Cell Signaling Technology (Danvers, MA, USA). α-tubulin (GTX112141), dynein (GTX80684), ubiquitin (GTX19247), ICAM (GTX100450), LC3B (GTX127375), acetyl-histone 2 (GTX633388) and histone 2 (GTX129418) were purchased from GeneTex (Hsinchu, Taiwan). PARP (sc-7150) were purchased from Santa Cruz (Island, CA, USA). VLA4 (11-0119-42) were purchased from eBioscience Inc. (San Diego, CA, USA). The labeled secondary antibodies were horseradish peroxidase (HRP)-conjugated anti-mouse or anti-rabbit IgG antibodies (Jackson ImmunoResearch Inc., West Grove, PA, USA).

### Cell Culture

RPMI-8226 and NCI-H929 were purchased from Bioresource Collection and Research Center (Hsinchu, Taiwan). The human bone marrow stromal cell line HS-5 was kindly provided by Prof. Yu, Alice Lin-Tsing (Genomics Research Center, Academia Sinica, Taipei, Taiwan). The cells were cultured in Roswell Park Memorial Institute medium (RPMI) 1640 (RPMI-82226 and NCI-H929) or Dulbecco's Modified Eagle's medium (DMEM) (HS-5), respectively supplemented with 20% (v/v) (RPMI-82226 and NCI-H929) and 10% (v/v) (HS-5) heat-inactivated fetal bovine serum (both from InvitrogenTM Life Technologies, Carlsbad, CA, USA), 100 U/mL of penicillin, 100 μg/mL of streptomycin, and 10 mM sodium pyruvate (Biological Industries, Kibbutz Beit Haemek, Israel). All cells were maintained at 37°C in a humidified atmosphere of 5% CO_2_ in air were periodically checked for Mycoplasma contamination. These cells have performed STR-PCR profiling at BCRC.

### Cell Cytotoxicity and Cell Proliferation Assay

Cell cytotoxicity was measured by the colorimetric MTT assay. Cells (1 × 10^5^) in 1 ml of medium in 24-well plates were incubated with vehicle (control) or vehicle with test compound for 48 h. After various treatments, 1 mg/mL of MTT was added and the plates were incubated at 37°C for an additional 2 h, then the cells were pelleted and lysed by 10%SDS with 0.01 M HCl, and the absorbance at 570 nm was measured on a microplate reader. Cells (1 × 10^4^) were incubated for 48 h with the indicated concentrations of test compound and the cell proliferation was measured by the 5-bromo-2′-deoxyuridine (BrdU) assay (Roche, Mannhein, Germany).

### Immunoblot and Immunoprecipitation Analyses

Cells (1 × 10^6^) were incubated for 10 min at 4°C in lysis buffer (20 mM HEPES, pH 7.4, 2 mM EGTA, 50 mM β-glycerophosphate, 0.1% Triton X-100, 10% glycerol, 1 mM DTT, 1 μg/mL of leupeptin, 5 μg/mL of aprotinin, 1 mM phenylmethylsulfonyl fluoride, and 1 mM sodium orthovanadate), were scraped off, incubated on ice for an additional 10 min, and centrifuged at 17, 000 g for 30 min at 4°C. Protein samples (80 μg) were then electrophoresed on sodium dodecyl sulfate polyacrylamide gels (SDS-PAGE) and transferred onto a nitrocellulose membrane, which was then blocked by incubation for 30 min at room temperature with 5% bovine serum albumin (BSA) in phosphate-buffered saline with 10% tween-20 (PBST). Immunoblotting was performed by overnight incubation at 4°C with primary antibodies in PBST, followed by incubation for 1 h at room temperature with HRP-conjugated secondary antibodies. Bound antibodies were measured using ECL reagent (Advansta Corp., Menlo Park, CA, USA) and exposure to photographic film. In the immunoprecipitation assay, cell lysates (100 μg) were immunoprecipitated overnight at 4°C with 1 μg of anti-ubiquitin or dynein antibody and A/G agarose beads. The precipitated beads were washed three times with 1 mL of ice-cold cell lysis buffer and bound immune complexes separated by 8% SDS-PAGE, followed by immunoblotting using the anti-HDAC6 antibody.

### Xenograft Studies

RPMI-8226 cells (1 × 10^7^) were implanted subcutaneously into eight-week-old male nude mice. When the tumors reached an average volume of 200 mm^3^, the mice were randomly divided into four groups (*n* = 5) and then were treated intraperitoneally with the vehicle (0.5% EtOH/0.5% Cremophor in 5% dextran, 0.2 mL/20 g mouse), BTZ (0.5 mg/kg, i.p., qd), MPT0G413 (25 mg/kg, i.p., qwk), or combination BTZ with MPT0G413 treatment. The length (L) and width (W) of the tumor were measured by caliper every 3 to 4 days, and the tumor volume was calculated as L × W^2^/2. The percentage of tumor growth inhibition (%TGI) as determined by the formula: {1 – [(Tt/T0)/(Ct/C0)]/1 – [C0/Ct]} × 100. Tt: tumor volume of treated at time t. T0: tumor volume of treated at time 0. Ct: tumor volume of control at time t. C0: tumor volume of control at time 0. Exclusion criteria and animal experiments were performed in accordance with relevant guidelines and regulations followed ethical standards, and protocols have been reviewed and approved by Animal Use and Management Committee of Taipei Medical University (IACUC no. LAC-2015-0163).

### Cell Adhesion Assay

RPMI-8226 or NCI-H929 (10^5^) were treated with MPT0G413, BTZ, or the combination therapy for 24 h, then the MM cells (5 × 10^4^) were labeled for 1 h at 37°C with 0.1 μg/mL BCECF-AM and washed twice with growth medium, and then, labeled cells were added to HS-5 and incubated for 1 h. Non-adherent cells were removed from the plates by phosphate-buffered saline (PBS) washed three times, and the adherent cells were photographed by Leica DMIRE2 inverted microscope. The numbers of adherent MM cells were counted in four randomly chosen fields per well at × 100 magnification in fluorescent microscopy.

### Quantification of VCAM-1 Expression

The level of cell surface VCAM-1 expression was determined by ELISA assay (28). RPMI-8226 or NCI-H929 (10^5^) were treated with MPT0G413, BTZ, or the combination therapy for 24 h, and then, MM cells were added to HS-5 and incubated for 1 h. Non-adherent cells were removed from the plates by phosphate-buffered saline (PBS) washed three times, the cells were washed twice with PBS and fixed at room temperature with 1% paraformaldehyde for 30 min. After washing with PBS, they were then blocked with 1% BSA in Tris-buffered saline containing 0.05% Tween-20 (TTBS) for 15 min before being incubated successively with anti-VCAM-1 antibody (1:100) for 1 h and horseradish peroxidase-labeled anti-mouse antibody (1:1000) for 30 min. After the incubation, the cells were washed twice with PBS. O-Phenylenediamine dihydrochloride substrate [0.4 mg/ml in phosphate-citrate buffer, pH 5.0; 24.3 mM citric acid; 51.4 mM Na2HPO4 · 12 H2O; 12% H2O2 (v/v)] was then applied to the cells for 30 min and 3 M sulfuric acid added to stop the reaction. The absorbance was measured at 450 nm by ELISA reader. Each assay was performed in triplicate.

### Immunofluorescence Assay

RPMI-8226 or NCI-H929 cells were cultured on tissue culture–treated glass slides with or without 2.5 μM ACY-1215 and/or 2.5 nM BTZ. After 24 h, cells were fixed by 100% methanol and permeabilized by PBST. After blocking in 0.5% BSA, cells were stained with anti-ubiquitin, LC3B antibody (GeneTex, Irvine, CA), VLA4 (Invitrogen, San Diego, USA) 1:100 for 1 h at room temperature. Cells were washed and incubated with Alexa Fluor 488 goat anti–mouse Ab (Ubiquitin) or FITC-conjugated goat anti-Rabbit antibody (LC3B) for 1 h. After subsequent washes, DAPI was added for 30 min. The slides were mounted with 90% glycerol and images were taken using a Nikon Ti-E microscope with 60 × Plan-Apo VC/NA 1.4 oil lens equipped with an Andor Clara camera.

### Immunohistochemical Analysis

Tissue sections (5 μm) were dewaxed and rehydrated. Antigen retrieval was done by autoclaving the slides in Trilogy solution (Cell Marque, Hot Springs, AR) at 121°C for 10 min. After blocking with 3% H_2_O_2_ and 5% fetal bovine serum, the slides were allowed to react with against Caspase 3, acetyl-α-tubulin (1:100, Cell Signaling) at 4°C overnight. The slides were then incubated with polymer-HRP reagent (Dako Cytomation, Glostrup, Denmark). The peroxidase activity was visualized with diamino-benzidine tetrahydroxychloride solution (DAKO). The sections were counterstained with hematoxylin. Dark brown nuclear staining was defined as positive and no staining was defined as negative.

### ELISA Assay

RPMI-8226 or NCI-H929 (1 × 10^4^) were co-cultured with HS-5 and treated with MPT0G413, bortezomib or the combination therapy 24 h. The medium was collected and assayed for IL-6 or VEGF using commercial kits (Invitrogene, San Diego, USA).

### Data Analysis

The data are expressed as the mean ± SEM and were analyzed using one-way ANOVA. When ANOVA showed significant differences between groups, Tukey's *post hoc* test was used to determine the pairs of groups showing statistically significant differences. A *p* < 0.05 was considered statistically significant.

## Results

### MPT0G413 Suppresses Growth, Induces Apoptosis, and Inhibits HDAC6 Activity in Multiple Myeloma Cells

**Figure 1:**
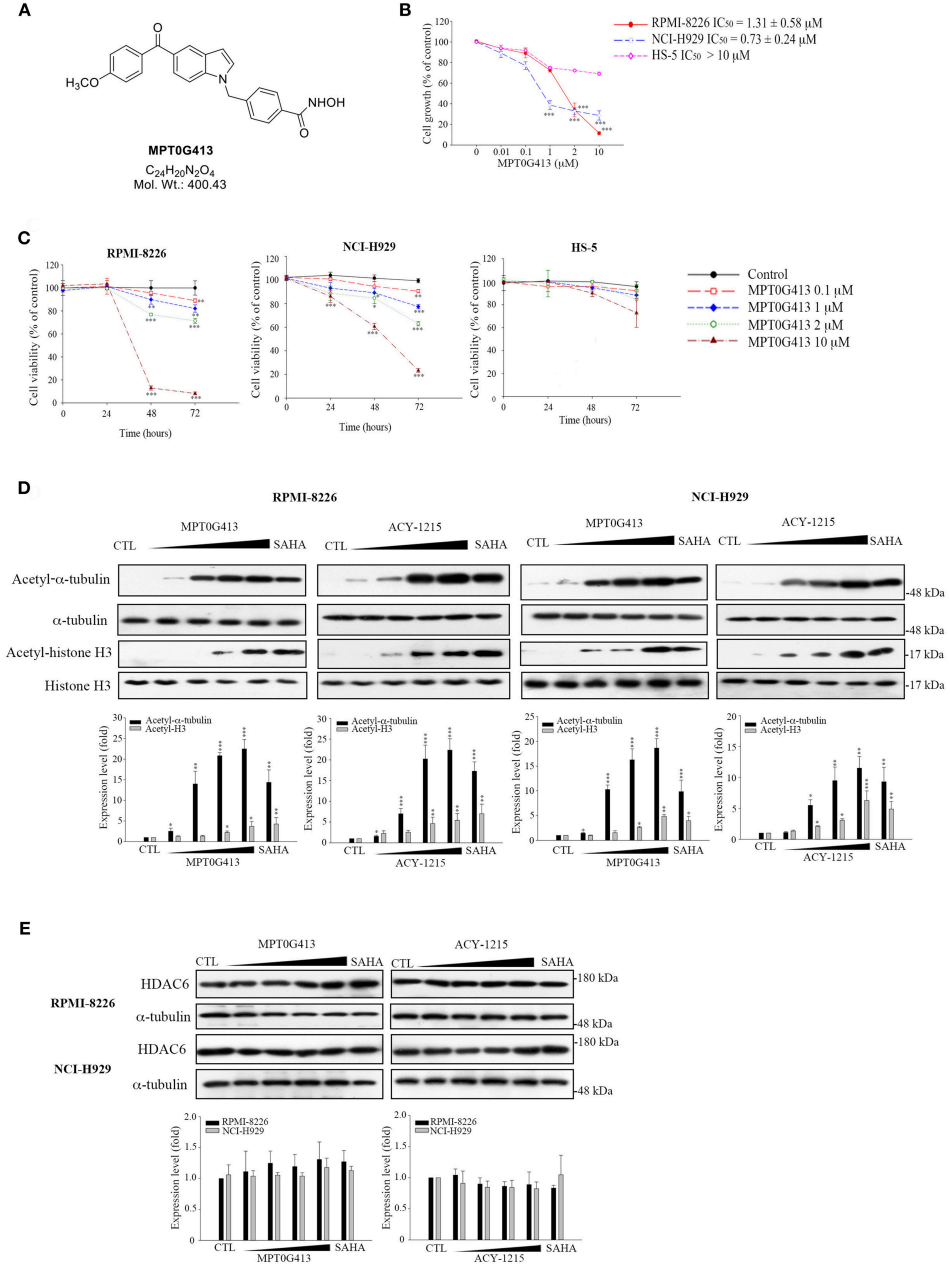
MPT0G413 potently inhibited histone deacetylase (HDAC6) and inhibited multiple myeloma cell growth and proliferation. **(A)** Chemical structure of MPT0G413. **(B)** Human multiple myeloma cell lines (RPMI-8226, NCI-H92; density, 1 × 10^4^) and human bone marrow stromal cells (HS-5; density, 5 × 10^3^) were incubated with or without the indicated concentrations of MPT0G413 for 48 h. Cell proliferation was evaluated using a 5-bromo-2′-deoxyuridine (BrdU) proliferation assay. **(C)** RPMI-8226, NCI-H929, and HS-5 cells were exposed to MPT0G413 at the indicated concentrations for 24, 48, and 72 h. Cell viability was measured using a MTT assay. **(D,E)** RPMI-8226 and NCI-H929 cells were treated with DMSO or MPT0G413 (0.1, 1, 2.5, 10 μM) and SAHA (2.5 μM) for 24 h. Cells were subsequently harvested, and the lysates were subjected to Western blotting of the indicated proteins. Protein levels in the Western blots were quantified using Image J software. The results are shown as mean ± SEM from three independent experiments. ^*^p < 0.05, ^**^p < 0.01, and ^***^p < 0.001, compared with HS-5 cells group **(B)** and the control group **(C–E)**.

The structure of MPT0G413 is shown in Figure 1A. In a previous study, we described the synthesis of this molecule and demonstrated its ability to inhibit HDAC6 with an IC_50_ value of 3.92 nM. Notably, MPT0G413 was 558.7-, 144.9-, 1058.7-, 164.8-, and 15255.1-fold more selective for HDAC6 than for HDAC1, 2, 3, 8, and 10, respectively (27). In this study, we first used a BrdU proliferation assay to examine whether MPT0G413 could inhibit the growth of MM cells. MPT0G413 was added to the cultures of two MM cell lines, RPMI-8226, NCI-H929, as well as to the BMSC line HS-5, and the GI_50_ values were evaluated. As shown in Figure 1B, MPT0G413 inhibited MM cell proliferation in a dose-dependent manner, with GI_50_ values of 1.31 ± 0.58 μM in RPMI-8226 and 0.73 ± 0.24 μM in NCI-H929; both values were lower than that in BMSCs (>10 μM), indicating the higher selectivity of MPT0G413 for MM cells. We also determined the cell cytotoxic effects of MPT0G413 using a MTT assay. The IC_50_ values of MPT0G413 were 4.15 ± 3.37 μM in RPMI-8226 and 11.20 ± 2.19 μM in NCI-H929, which were lower than the values for the HDAC6 inhibitors ACY-1215 (6.77 ± 2.38 and >100 μM, respectively) and Tubastatin A (13.00 ± 0.65 and > 100 μM, respectively) (Table 1, Supplemental Figure 1). In HS-5, all three drugs had an IC_50_ >100 μM. Furthermore, MPT0G413 inhibited MM cell viability in a dose- and time-dependent manner until 72 h, and this effect was less pronounced in HS-5 cells (Figure 1C). In summary, MPT0G413 appears to inhibit MM cell proliferation and viability and to specifically target malignant tumor cells.

**Table 1:** IC_50_ values (μM) of histone deacetylase (HDAC) inhibitors in multiple myeloma and bone marrow stromal cell (BMSC) lines.

	**RPMI-8226**	**NCI-H929**	**HS-5**
MPT0G413	4.15 ± 3.37	11.20 ± 2.19	>100
ACY-1215	6.77 ± 2.38	>100	>100
Tubastatin A	13.00 ± 0.65	>100	>100

We next determined the inhibitory effects of MPT0G413 on the activity of HDACs. To understand this effect in MM cells, we used Western blotting to analyze the accumulation of acetyl-α-tubulin in cell lysates. As shown in Figure 1D, MPT0G413 significantly increased the accumulation and acetylation of α-tubulin, the cytoplasmic HDAC6 substrate, in a concentration-dependent manner, but had little effect on the acetylation of histone H3K9, the nuclear substrate for class I HDACs. Furthermore, MPT0G413 treatment further enhanced acetyl-α-tubulin accumulation, compared to ACY-1215 treatment (1 μM). Further, significant acetyl-α-tubulin increase in response to lower concentration (0.1 μM) treatment in RPMI-8226 and NCI-H929 cells; MPT0G413 only induced mild acetyl-histone H4 levels increase at higher concentration (2.5 μM) in RPMI-8226 cells, and it even didn't cause significant acetyl-histone H4 increasing from 0.1 to 10 μM in NCI-H929 cells (Supplemental Figure 2). In addition, MPT0G413 and the pan-HDAC inhibitor SAHA induced similar levels of acetyl-α-tubulin accumulation; however, the former caused less H3K9 acetylation at the same concentration (2.5 μM). We further confirmed that the HDAC6 inhibition mediated by MPT0G413 was not due to a decrease in HDAC6 levels in either RPMI-8226 or NCI-H929 cells (Figure 1E). These results suggest that MPT0G413 is a potent HDAC6 selective inhibitor.

### Combination of MPT0G413 and Bortezomib Synergistically Enhanced Cell Apoptosis in Human Multiple Myeloma Cells

**Figure 2:**
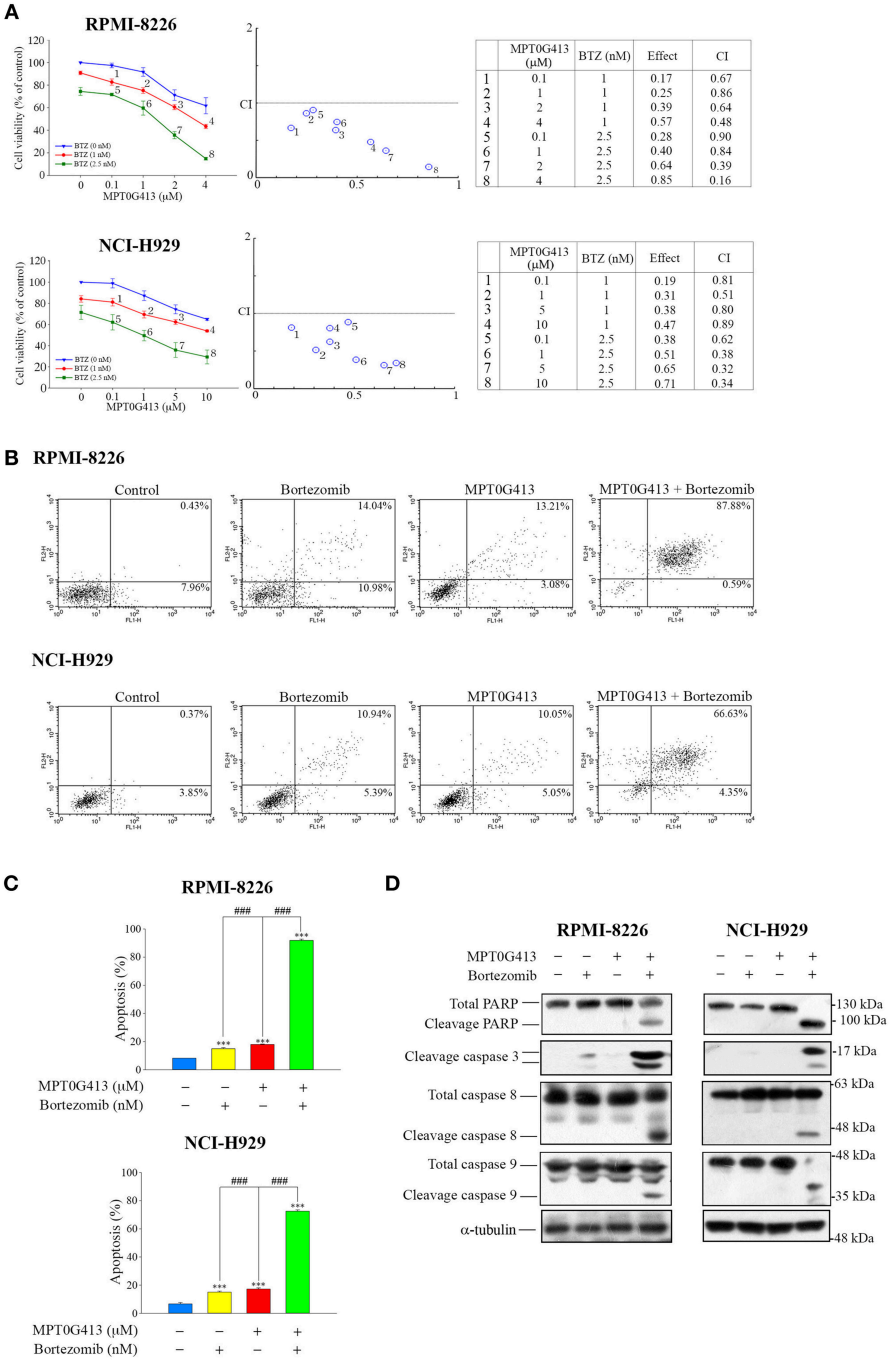
A combination of MPT0G413 and bortezomib synergistically inhibited multiple myeloma cell viability. **(A)** RPMI-8226 and NCI-H929 cells were treated with the indicated concentrations of MPT0G413, bortezomib, or both agents for 48 h. The effects on cell viability were analyzed using a MTT assay (left panel). The combination index values for the combination of MPT0G413 and bortezomib were calculated using CompuSyn software (middle and right panels). **(B)** RPMI-8226 and NCI-H929 cells were treated with MPT0G413 (4 μM in RPMI-8226 cells and 10 μM in NCI-H929 cells), bortezomib (2.5 nM), or combination therapy for 48 h, followed by staining with propidium iodide and annexin V and analysis by flow cytometry. **(C)** Proportions of apoptotic cells in response to the drug treatments described in **(B)**. Results are shown as mean ± SEM from three independent experiments. ^***^p < 0.001 compared with the control group. ###*p* < 0.001 compared with the relevant control group. **(D)** RPMI-8226 and NCI-H929 cells were treated with MPT0G413 (2.5 μM), bortezomib (2.5 nM), or combination therapy for 12 h. Whole cell lysates were immunoblotted with the indicated antibodies.

As a combination of ACY-1215 and BTZ had been used in a clinical trial of MM (25, 29), we further evaluated the ability of a combination of MPT0G413 and BTZ to induce apoptosis in MM cells. RPMI-8226 and NCI-H929 cells were incubated with increasing concentrations of MPT0G413 and BTZ 48 h, after which viability was assayed using a MTT assay. As shown in Figure 2A (middle and right panels), a significant decrease in viability was observed after combined treatment relative to single-agent therapy, and this combination effect was synergistic because the combined index values were <1.0. To characterize the mechanism of synergistic cytotoxicity induced by the combined treatment, we used annexin V and propidium iodide double-staining and flow cytometry to examine cell apoptosis. Notably, we observed significant increases in both early and late apoptosis (88.47% in RPMI-8226 and 70.98% in NCI-H929) after 48 h of treatment with the combination therapy, whereas either BTZ (2.5 nM) or MPT0G413 (4 μM in RPMI-8226 and 10 μM in NCI-H929) alone only induced mild apoptosis (Figures 2B,C). Furthermore, the combination of MPT0G413 and BTZ for 24 h caused marked increases in the levels of cleaved PARP, caspase 3, caspase 8, and caspase 9 (Figure 2D) in both RPMI-8226 and NCI-H929 cells, further indicating the synergistic effect of this combination therapy on apoptosis.

### MPT0G413 Disrupted Bortezomib-Induced Aggresome Formation by Inhibiting HDAC6/Dynein Binding

**Figure 3:**
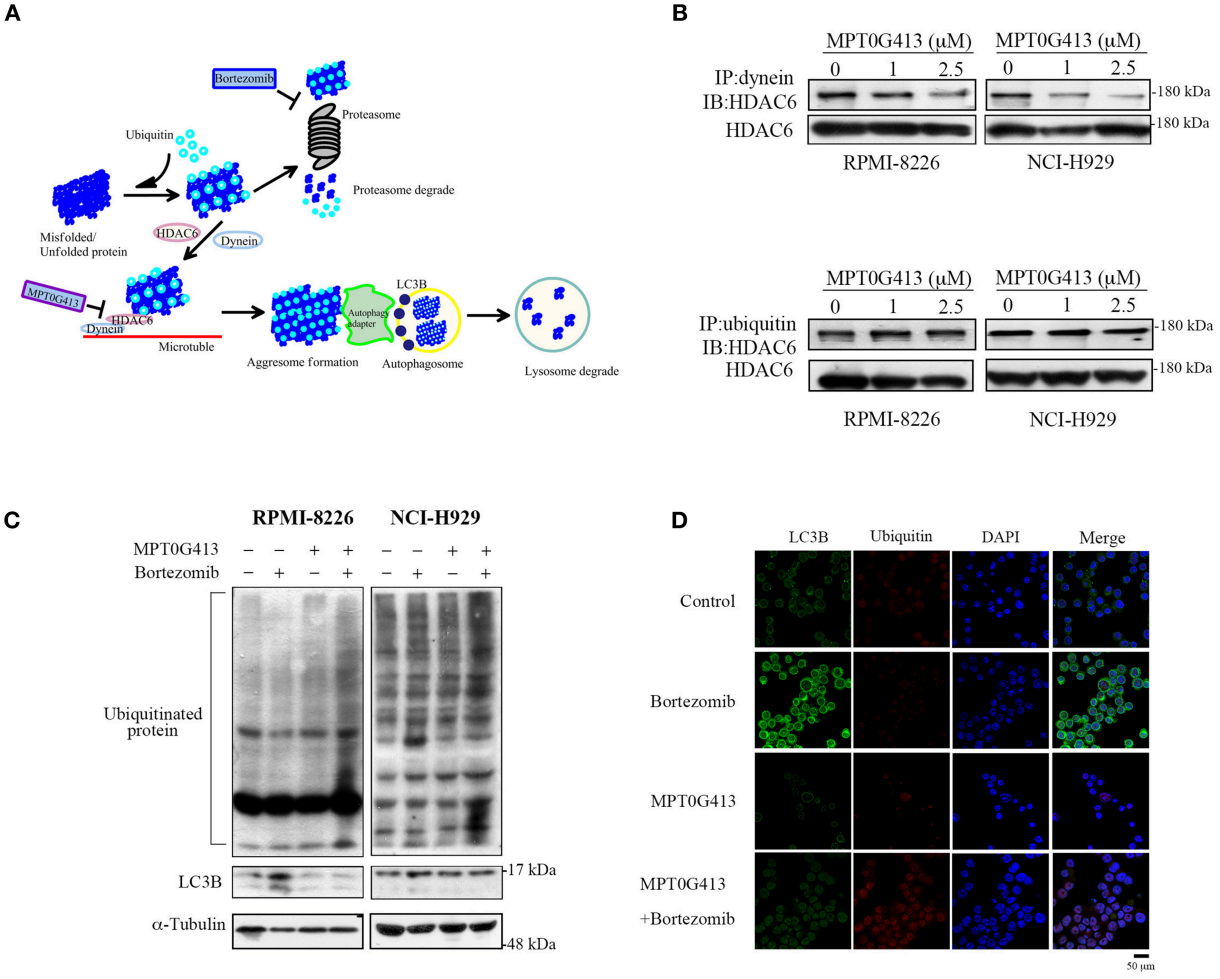
MPT0G413 inhibited the binding of histone deacetylase (HDAC6) to dynein and induced the significant accumulation of polyubiquitinated proteins when combined with bortezomib. **(A)** Hypothetical rationale of proteasome and aggresome pathway inhibition by bortezomib and MPT0G413. **(B)** RPMI-8226 and NCI-H929 cells were treated with MPT0G413 (1 and 2.5 μM) for 6 h, and total cell lysates were subjected immunoprecipitation with 1 μg of an anti-dynein or anti-ubiquitin antibody, followed by immunoblotting with an anti-HDAC6 antibody. **(C)** RPMI-8226 and NCI-H929 cells were cultured with MPT0G413 (2.5 μM), bortezomib (2.5 nM), or combined therapy for 12 h. Whole cell lysates were subjected to Western blotting with antibodies specific for ubiquitin and LC3. **(D)** NCI-H929 cells were treated with MPT0G413 (2.5 μM), bortezomib (2.5 nM), or combination therapy for 6 h and stained by anti-LC3B (green) and anti-ubiquitin (red) antibodies and DAPI (blue) prior to confocal microscopy analysis. Scale bar = 50 μm.

Previous studies have demonstrated that ubiquitinylated unfolded proteins are targeted for degradation by both the proteasomal and aggresomal pathways (29). In this study, we hypothesized that the combination of MPT0G413 and BTZ would block both pathways and trigger a significant accumulation of polyubiquitinated proteins, leading increases in cellular stress and apoptosis (Figure 3A). HDAC6 contains both dynein motor binding and ubiquitin binding domains, and a previous study revealed that this enzyme could bind polyubiquitinated proteins, recruit these unfolded/misfolded proteins to dynein motors along microtubules, and thus enhance degradation via the autophage-lysosomal pathway (24). Furthermore, BTZ treatment was shown to enhanced autophagosome formation, as evidenced by an increase in the levels of the autophagosomal marker LC3B (30). We first examined whether MPT0G413 inhibited HDAC6 activity via the ubiquitin binding domain or dynein binding domain. After treating RPMI-8226 and NCI-H929 cells with MPT0G413 (1 and 2.5 μM) for 12 h, the co-immunoprecipitation of dynein with HDAC6 was markedly inhibited in a dose-dependent manner, whereas the co-immunoprecipitation of ubiquitinated proteins and HDAC6 was unaffected (Figure 3B). Moreover, the combination of MPT0G413 and BTZ increased the accumulation of polyubiquitinated proteins in RPMI-8226 and NCI-H929 cells when compared with either agent alone (Figure 3C). To further confirm the effects of MPT0G413 inhibited aggresome formation, we treated NCI-H929 cells with 2.5 μM MPT0G413 and/or 2.5 nM BTZ for 12 h, followed by staining with an anti-LC3B and/or anti-ubiquitin antibody. Notably, cells treated with BTZ expressed LC3B in the cytoplasm, suggesting the inhibition of the proteasomal pathway and an increase in aggresome formation in the cell (Figures 3C,D). The combination of MPT0G413 and BTZ, which blocked the proteasomal and aggresomal pathways, led to an obvious increase in the expression of ubiquitinated proteins but relatively less aggresome formation (LC3B) (Figures 3C,D). These data indicate that the combination therapy inhibited both the proteasomal and aggresomal pathways and induced the aggregation of polyubiquitinated proteins in MM cells, leading to apoptosis (Figures 2B,C). Our data thus supported the synergistic anti-MM activity of this combination of MPT0G413 and BTZ.

### Combination of MPT0G413 and Bortezomib Inhibited Multiple Myeloma Cell Growth in RPMI-8226 Xenograft Mice

**Figure 4:**
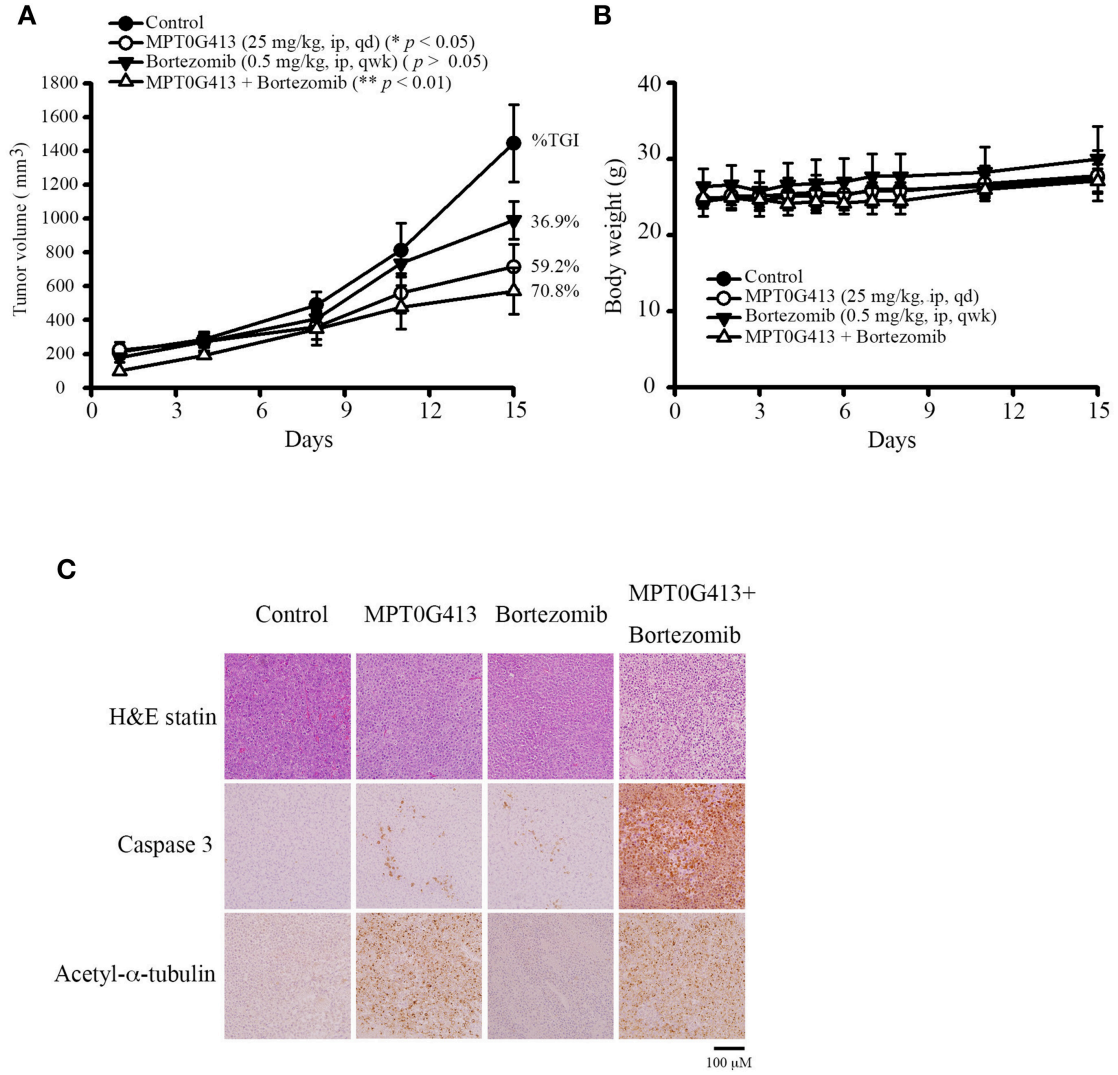
Anticancer activities of MPT0G413 alone and combined with bortezomib in a RPMI-8226 xenograft model**. (A,B)** Nude mice bearing RPMI-8226 tumors (~200 mm^3^) were divided into four groups and treated with saline, MPT0G413 (25 mg/kg), bortezomib (0.5 mg/kg), or a combination of MPT0G413 and bortezomib for 15 days. Tumor volumes **(A)** and body weights **(B)** were measured. Results are shown as mean ± SEM (n = 5). **(C)** Tumor was excised from each mouse after a 15-day treatment. Paraffin sections of RPMI-8226 xenografts were stained with hematoxylin and eosin and antibodies specific for cleaved caspase 3 or acetyl-α-tubulin. Sections were examined by light microscopy (200 × magnification). Scale bar = 100 μm.

We next evaluated the ability of MPT0G413 to inhibit *in vivo* tumor growth in a xenograft mouse model. Upon reaching a tumor size of 200 mm^3^, mice received intraperitoneal injections of vehicle (control), MPT0G413 (25 mg/kg), BTZ (0.5 mg/kg), or a combination of MPT0G413 and BTZ for 15 days or until the endpoint tumor volume of 1,500 mm^3^ was achieved. As shown in Figure 4A, the administration of either MPT0G413 or BTZ alone significantly reduced the tumor volume, with tumor growth inhibition (TGI) rates of 36.9 and 59.2%, respectively. Notably, the combination therapy led to a more potent reduction in tumor volume, with a TGI of 70.8%. No significant body weight losses were observed during any of the treatment periods (Figure 4B). H&E staining of the tumor tissue revealed that the combination therapy enhanced nuclear condensation, while immunohistochemical staining revealed more apoptotic cells and increased cleaved caspase 3 expression in response to combination treatment. Moreover, slides from the MPT0G413 treatment group revealed the accumulation of acetyl-α-tubulin (Figure 4C). These results demonstrated that the combination of MPT0G413 and BTZ could induce MM cell apoptosis *in vivo*.

### Combination of MPT0G413 and Bortezomib Inhibited the Adhesion of Multiple Myeloma Cells to Bone Marrow Stromal Cells

**Figure 5:**
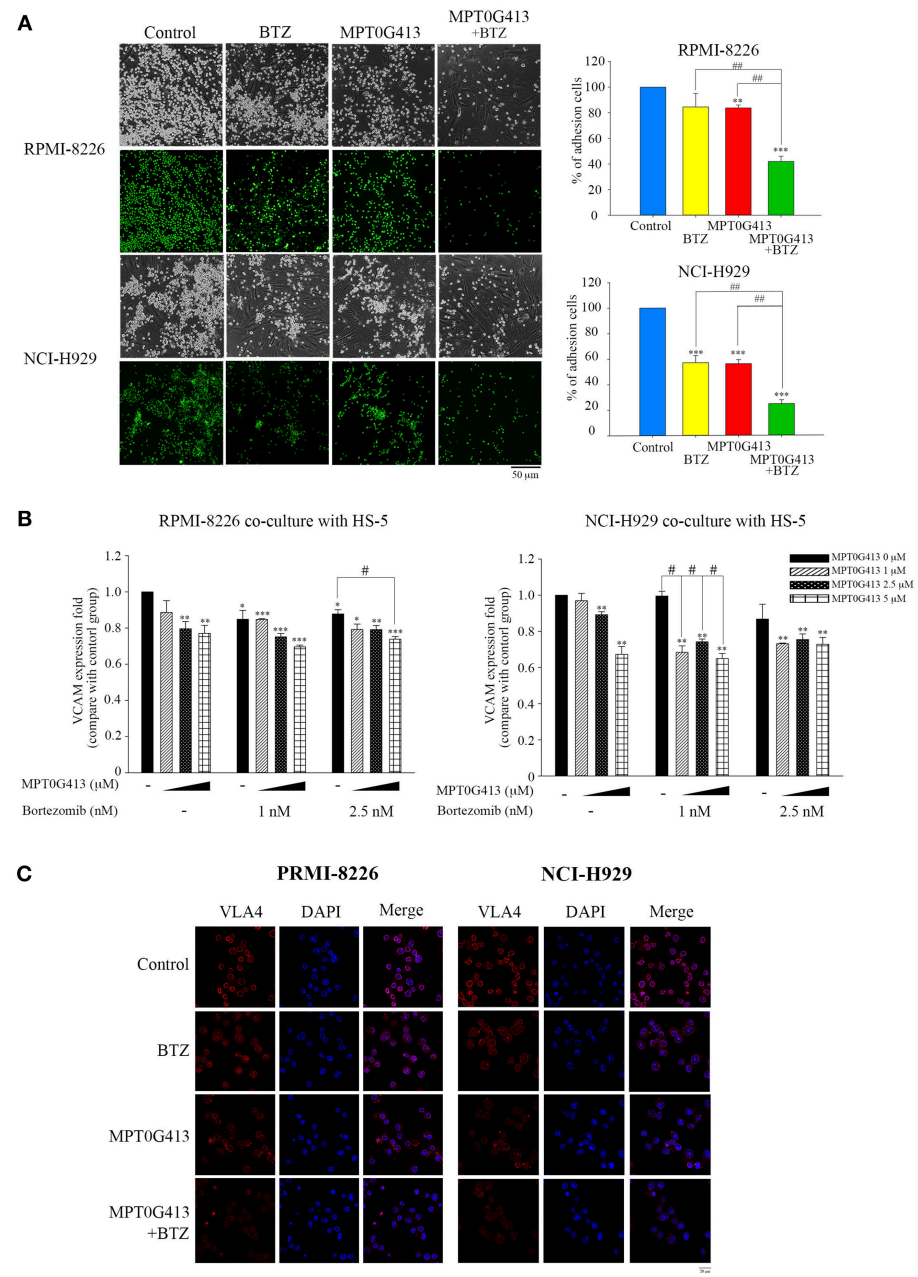
A combination of MPT0G413 and bortezomib inhibited the adhesion of multiple myeloma cells to bone marrow stromal cells. **(A)** RPMI-8226 and NCI-H929 multiple myeloma cells were treated with MPT0G413 (2.5 μM), bortezomib (2.5 nM), or both agents for 24 h and subsequently labeled with 10 μM BCECF-AM. The labeled cells were subsequently co-cultured with HS-5 bone marrow stromal cells for 2 h. After incubation, non-adherent cells were removed by gentle washing with phosphate-buffered saline, and adherent multiple myeloma cells were, respectively photographed by light and fluorescence microscopy. Images depict 40 × magnification (Scale bar = 50 μm). Image J software was used to quantify the fluorescence microscopy images. **(B)** Multiple myeloma cells were co-cultured with HS-5 cells and incubated with MPT0G413 (2.5 μM), bortezomib (2.5 nM), or a combination of both agents for 24 h. The expression of VCAM-1 was measured by ELISA as described in the Materials and Methods. Results represent the mean ± SEM from three independent experiments. ^*^p < 0.05, ^**^p < 0.01, and ^***^p < 0.001 compared with the control group. #*p* < 0.05 and ##*p* < 0.01 compared with the relevant control group. **(C)** RPMI-8226 and NCI-H929 cells were treated with MPT0G413 (2.5 μM), bortezomib (2.5 nM), or combination therapy for 6 h. Subsequently, the samples were stained with anti-VLA4 (red) and DAPI (blue) prior to a confocal microscopy analysis. Scale bar = 20 μm.

Previous studies demonstrated that the adherence of MM cells to BMSC cells stimulates the latter to release growth factors (e.g., VEGF and IL-6) that would support the proliferation and survival of the former cell type (31, 32). In this study, we evaluated the effects caused by the exposure of MM cells in a BM microenvironment to combination therapy. First, we studied whether a combination of MPT0G413 and BTZ could inhibit the adherence of MM cells to BMSCs. As shown in Figure 5A, the combination therapy significantly inhibited this adherence, compared with either single-agent therapy. We further assessed the expression of adhesion molecules, such as vascular cell adhesion molecule-1 (VCAM-1) and VLA-4, using ELISA and immunofluorescence imaging, respectively and found that the combination of MPT0G413 and BTZ decreased the expression of both proteins (Figures 5B,C, respectively) in HS-5 and MM cells.

**Figure 6:**
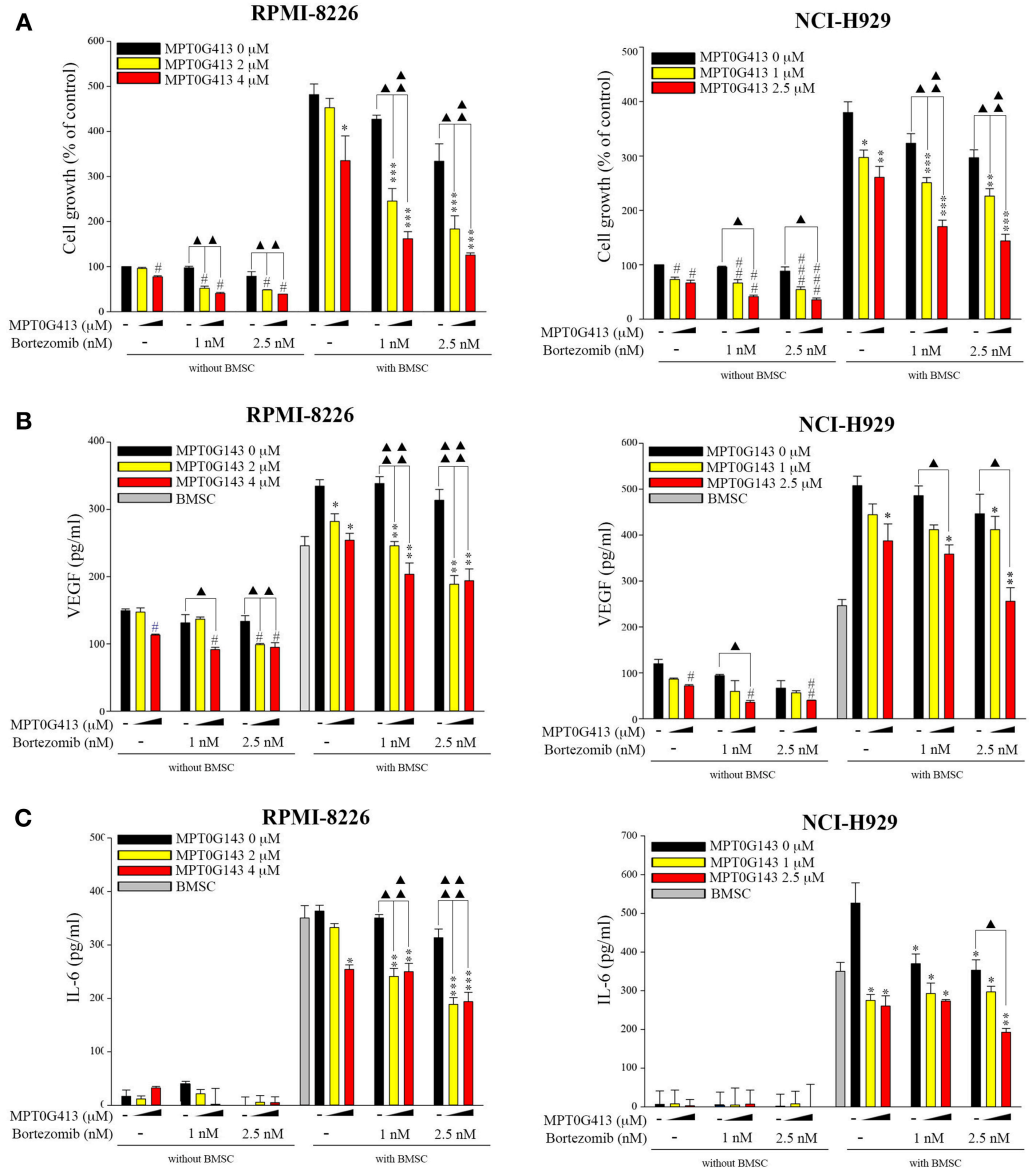
Combination treatment of MPT0G413 and bortezomib inhibited multiple myeloma cell growth and VEGF and IL-6 release. **(A–C)** RPMI-8226 and NCI-H929 multiple myeloma cells were co-cultured with or without HS-5 bone marrow stromal cells and subsequently treated with the indicated concentrations of MPT0G413, bortezomib, or both therapies for 48 h. Cell proliferation **(A)** was evaluated using a BrdU proliferation assay. VEGF **(B)** and IL-6 **(C)** levels were detected using respective ELISA kits. #*p* < 0.05, ##*p* < 0.01, and ###*p* < 0.001 compared with the untreated group (without HS-5). ^*^p < 0.05, ^**^p < 0.01, and ^***^p < 0.001 compared with the untreated group (with HS-5) (*n* = 3).

**Figure 7:**
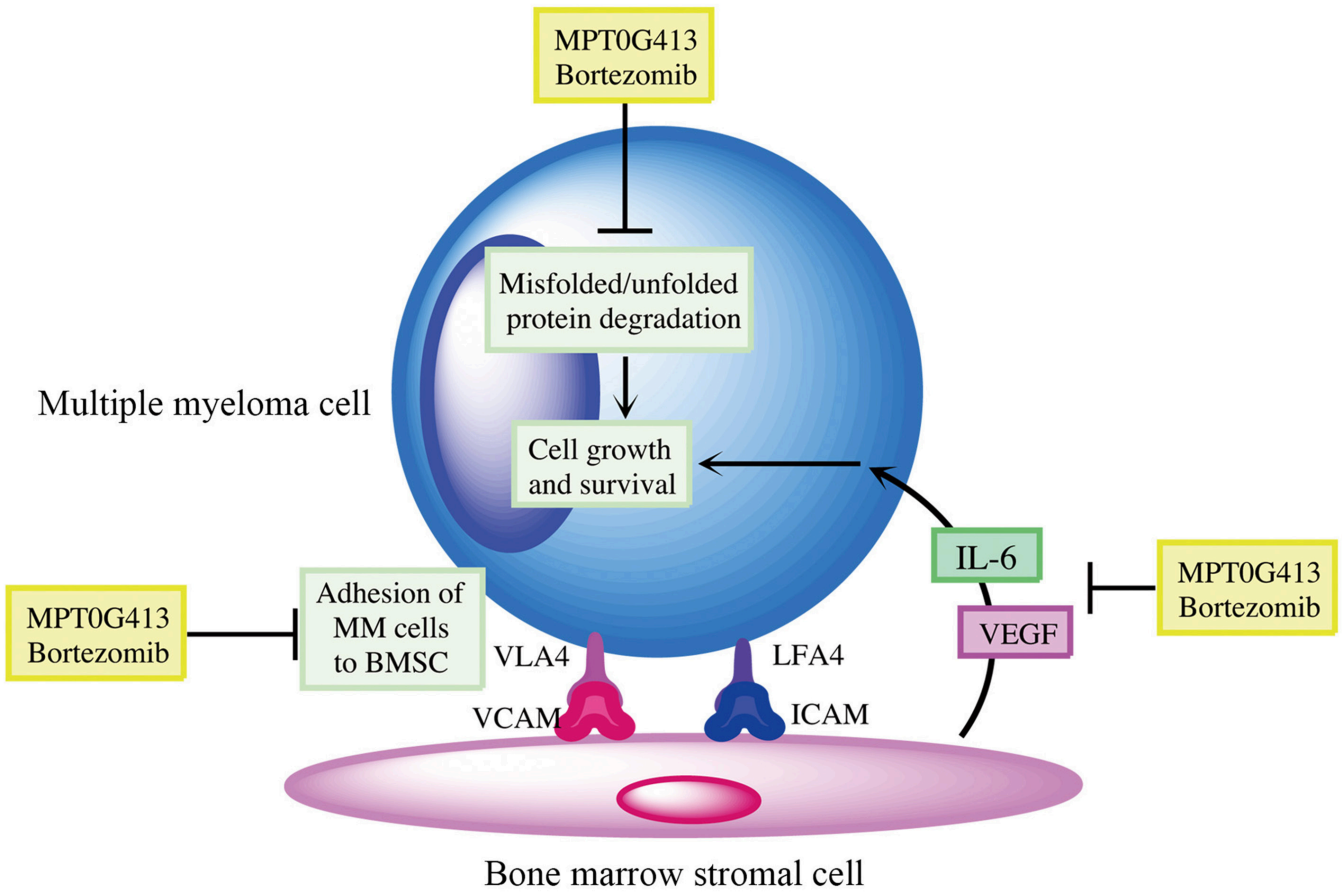
Summary of mechanisms by which MPT0G413 and bortezomib inhibit multiple myeloma cell survival and growth.

We then examined whether the combination of MPT0G413 and BTZ could inhibit MM cell growth in the presence of BMSCs after 48 h incubation. Using a BrdU assay, we found that the combination therapy not only significantly inhibited MM cell growth relative to either single-agent therapy in the absence of BMSCs, but also markedly downregulated MM cell proliferation in the co-culture system (Figure 6A). Moreover, the combination therapy reduced VEGF and IL-6 levels in the BM microenvironment (Figures 6B,C). In summary, the combination of MPT0G413 and BTZ inhibited the adherence of MM cells to BMSCs, and suppressed cell growth and VEGF and IL-6 secretion in the bone marrow microenvironment. Figure 7 summarizes the mechanisms suggested to underlie these observed effects.

## Discussion

HDACs, which regulate the expression of tumor-associated genes via histone modification, play crucial roles in tumorigenesis. Accordingly, these enzymes have been recognized as therapeutic targets in both solid tumors and hematologic malignancies (33–35). To date, several small-molecule inhibitors of HDACs have been introduced for the treatment of several hematologic malignancies. For example, SAHA, PXD101, and LBH589 have been approved for the treatment of cutaneous T-cell lymphoma, peripheral T-cell lymphoma, and MM, respectively (36). According to previous studies, the combination of an HDAC6 inhibitor and BTZ synergistically induced apoptosis in MM cells via inhibition of the proteasomal and aggresomal pathways of protein degradation and subsequent activation of apoptosis pathways (1, 25, 30). Moreover, findings from a clinical study of a combination of an HDAC6 inhibitor and BTZ for the treatment of MM have been encouraging (37). These findings indicated that HDAC6 is a suitable target for MM treatment. HDAC6 is also suitable for research purposes, as knockout mice are viable and develop normally (19). Previously, we identified MPT0G413 as a potent and highly selective HDAC6 inhibitor (27). In this study, we evaluated whether MPT0G413 could exert anti-MM activity. Our results showed that MPT0G413 treatment markedly inhibited the growth and viability of MM cells but did not similarly inhibit the growth of normal BMSCs (Figures 1B,C). In other words, MPT0G413 appears selective for tumor cells.

To understand the specific inhibitory effect of MPT0G413 on HDAC6 activity, we evaluated the effect of this drug on the acetylation of histones, as well as α-tubulin, a cytosolic HDAC6 substrate (38). Notably, we found that MPT0G413 induced α-tubulin acetylation at low concentrations but only triggered lysine acetylation on histone H3 at higher concentrations. Furthermore, we demonstrated that the specific inhibitory effect of MPT0G413 on HDAC6 was not mediated by a decrease in HDAC6 protein levels (Figure 1E).

We further observed a synergistic increase in MM cell apoptosis (Figure 2) and ubiquitinated protein accumulation with the combination of MPT0G413 and BTZ, compared to either single-agent therapy (Figures 3C,D) and determined that this effect was mediated by the inhibition of the proteasomal and aggresomal pathways. HDAC6 contains both ubiquitin and dynein motor binding domains and can thus bind to polyubiquitinated misfolded proteins and dynein, respectively. Consequently, HDAC6 transports its misfolded protein cargo along microtubules to the aggresomes, where recognition by autophagy adapter proteins mediates the clearance of the aggregate via the autophagy-lysosomal pathway (24, 39, 40). As shown in Figure 3B, MPT0G413 blocked the binding of HDAC6 with dynein and thus, inhibited the transport of misfolded proteins transport to the aggresome. To confirm that HDAC6 inhibition disrupted aggresome formation (41), we evaluated the expression and localization of LC3B, an autophagosomal marker (40), using Western blotting and immunofluorescence imaging. Notably MPT0G413 treatment significantly disrupted aggresome formation, increased polyubiquitinated protein accumulation (Figures 3C,D), and induced MM cell apoptosis (Figures 2B–D).

We also evaluated the anti-MM effect of MPT0G413 by *in vivo* model. Here MPT0G413 significantly inhibited tumor growth, and the combination treatment further suppressed tumor growth with a %TGI of 70.8%. Notably, none of the treatments cause body weight losses (Figures 4A,B). Further, MPT0G413 even at high dose (1,000 mg/kg/day) by intraperitoneal injection for 6 days still does not cause body weight loss in mice (27). In contrast, previous studies demonstrated that ACY-1215 along only decreased around 30% tumor volume in mice (25); and ACY-1215 alone or combination with bortezomib within the first 5–10 days caused body weight loss >10% in mice (42). Panobinostat, a pan-HDAC inhibitor, also caused body weight loss in mice, and the MTD of panobinostat was 20 mg/kg (43). The immunohistochemical analysis also revealed that MPT0G413 increased the accumulation of acetyl-α-tubulin when administered alone and significantly increased the level of cleaved caspase 3 in combination with bortezomib (Figure 4C), and there was no obvious increasing acetyl-Histone H2, acetyl-Histone H3 and acetyl-Histone H4 levels between MPT0G413 vs. control group (Supplemental Figure 3). These results clearly translate our cellular findings to an established animal models of MM.

In the bone marrow microenvironment, the binding of MM cells to BMSCs triggers the secretion of cytokines such as VEGF and IL-6 to promote MM cell growth, survival, migration, and chemotherapeutic resistance (1, 44). Previously, HDAC6 inhibition significantly compromised the migration and adhesion of Burkitt's lymphoma cells (45) and reduced VLA4 expression in hematopoietic stem cells and acute myeloid leukemia blast cells (46). BTZ was also shown to downregulate VLA4 expression and overcome cell adhesion-mediated drug resistance (47). We hypothesized that a combination therapy would suppress the adhesion of MM cells to BMSC via VLA4 and VCAM-1. Using a MM/BMSC co-culture model to mimic the bone marrow microenvironment, both MPT0G413 and bortezomib inhibited the adherence of MM cells to BMSC (Figure 5A), while combination treatment further enhanced this effect by blocking the connection between VLA4 and VCAM-1. In our previous study, HDAC6 inhibition mediated Hsp90 acetylation, which caused HIF-1α degradation and subsequently, VEGF downregulation (48). In this study, we observed similar reductions in VEGF, as well as IL-6, secretion from BMSC in response to MPT0G413. These data reasonably explain why our combination treatment synergistically inhibited the growth of MM cells.

In conclusion, both *in vitro* and *in vivo* studies strongly suggest that MPT0G413 and bortezomib synergistically act via the simultaneous inhibition of the aggresomal and proteasomal pathways, respectively, to enhance MM cytotoxicity. Our findings warrant the advancement of this combination therapy into clinical development.

## Ethics Statement

Exclusion criteria and animal experiments were performed in accordance with relevant guidelines and regulations and followed ethical standards, and protocols have been reviewed and approved by Animal Use and Management Committee of Taipei Medical University (IACUC no. LAC-2015-0163).

## Author Contributions

F-IH, S-LP, and C-RY conceived the research. F-IH conducted the *in vitro* experiments, analyzed the data, and wrote the manuscript draft. C-RY edited the manuscript. Y-WW and T-YS conducted the *in vivo* experiments. S-LP interpreted the data. J-PL and M-HL synthesized the compound. S-LP and C-RY supervised the study. All authors reviewed the manuscript.

### Conflict of Interest Statement

The authors declare that the research was conducted in the absence of any commercial or financial relationships that could be construed as a potential conflict of interest.

## References

[1] Hideshima T Mitsiades C Tonon G Richardson PG Anderson KC. Understanding multiple myeloma pathogenesis in the bone marrow to identify new therapeutic targets. Nat Rev Cancer. (2007) 7:585–98. 10.1038/nrc2189 17646864

[2] Siegel RL Miller KD Jemal A Cancer Statistics, 2018. CA Cancer J Clin. (2018) 68:7–30. 10.3322/caac.21442 29313949

[3] San Miguel JF Schlag R Khuageva NK Dimopoulos MA Shpilberg O Kropff M Bortezomib plus melphalan and prednisone for initial treatment of multiple myeloma. N Engl J Med. (2008) 59:906–17. 10.1056/NEJMoa0801479 18753647

[4] Kumar SK Lee JH Lahuerta JJ Morgan G Richardson PG Crowley J. Risk of progression and survival in multiple myeloma relapsing after therapy with IMiDs and bortezomib: a multicenter international myeloma working group study. Leukemia. (2012) 26:149–57. 10.1038/leu.2011.196 21799510 PMC4109061

[5] Laubach J Garderet L Mahindra A Gahrton G Caers J Sezer O. Management of relapsed multiple myeloma: recommendations of the international myeloma working group. Leukemia. (2016) 30:1005–17. 10.1038/leu.2015.356 26710887

[6] Mitsiades N Mitsiades CS Richardson PG McMullan C Poulaki V Fanourakis G. Molecular of histone deacetylase inhibition in human malignant B cells. Blood. (2003) 101:4055–62. 10.1182/blood-2002-11-3514 12531799

[7] Jagannath S Dimopoulos MA Lonial S. Combined proteasome and histone deacetylase inhibition: A promising synergy for patients with relapsed/refractory multiple myeloma. Leuk Res. (2010) 34:1111–8. 10.1016/j.leukres.2010.04.001 20472288

[8] Maiso P Carvajal-Vergara X Ocio EM López-Pérez R Mateo G Gutiérrez N. The histone deacetylase inhibitor LBH589 is a potent antimyeloma agent that overcomes drug resistance. Cancer Res. (2006) 66:5781–9. 10.1158/0008-5472.CAN-05-4186 16740717

[9] Hideshima T Qi J Paranal RM Tang W Greenberg E West N. Discovery of selective small-molecule HDAC6 inhibitor for overcoming proteasome inhibitor resistance in multiple myeloma. Proc Natl Acad Sci USA. (2016) 113:13162–7. 10.1073/pnas.1608067113 27799547 PMC5135369

[10] Piekarz R Bates S. A review of depsipeptide and other histone deacetylase inhibitors in clinical trials. Curr Pharm Des. (2004) 10:2289–98. 10.2174/1381612043383980 15279609

[11] Richardson PG Harvey RD Laubach JP Moreau P Lonial S San-Miguel JF. Panobinostat for the treatment of relapsed or relapsed/refractory multiple myeloma: pharmacology and clinical outcomes. Expert Rev Clin Pharmacol. (2016) 9:35–48. 10.1586/17512433.2016.1096773 26503877

[12] Srinivas NR. Clinical pharmacokinetics of panobinostat, a novel histone deacetylase (HDAC) inhibitor: review and perspectives. Xenobiotica. (2017) 47:354–68. 10.1080/00498254.2016.1184356 27226420

[13] Marks PA Xu WS. Histone deacetylase inhibitors: potential in cancer therapy. J Cell Biochem. (2009) 107:600–8. 10.1002/jcb.22185 19459166 PMC2766855

[14] Dimopoulos M Siegel DS Lonial S Qi J Hajek R Facon T. Vorinostat or placebo in combination with bortezomib in patients with multiple myeloma (VANTAGE 088): a multicentre, randomised, double-blind study. Lancet Oncol. (2013) 14:1129–40. 10.1016/S1470-2045(13)70398-X 24055414

[15] Li Y Shin D Kwon SH Histone decetylase 6 plays role as a distinct regular of diverse cellular processes. FEBS J. (2013) 280:775–93. 10.1111/febs.12079 23181831

[16] Boyault C Gilquin B Zhang Y Rybin V Garman E Meyer-Klaucke W. HDAC6-p97/VCP controlled polyubiquitin chain turnover. EMBO J. (2006) 25:3357–66. 10.1038/sj.emboj.7601210 16810319 PMC1523186

[17] Hook SS Orian A Cowley SM Eisenman RN. Histone deacetylase 6 binds polyubiquitinthrough its zinc finger (PAZ domain) and copurifieswith deubiquitinating enzymes. Proc Natl Acad Sci USA. (2009) 99:13425–30. 10.1073/pnas.172511699 12354939 PMC129689

[18] Seigneurin-Berny D Verdel A Curtet S Lemercier C Garin J Rousseaux S Identification of components of the murine histonedeacetylase 6 complex: link between acetylation andubiquitination signaling pathways. Mol Cell Biol. (2001) 21:8035–44. 10.1128/MCB.21.23.8035-8044.2001 11689694 PMC99970

[19] Zhang Y Kwon S Yamaguchi T Cubizolles F Rousseaux S Kneissel M. Mice lacking histone deacetylase 6 have hyperacetylated tubulin but are viable and develop normally. Mol Cell Biol. (2008) 28:1688–701. 10.1128/MCB.01154-06 18180281 PMC2258784

[20] Yan J. Interplay between HDAC6 and its interacting partners: essential roles in the aggresome-autophagy pathway and neurodegenerative diseases. DNA Cell Biol. (2014) 33:567–80. 10.1089/dna.2013.2300 24932665

[21] Adams J. The proteasome: a suitable antineoplastic target. Nat Rev Cancer. (2004) 4:349–60. 10.1038/nrc1361 15122206

[22] Hideshima T Richardson PG Anderson KC. Mechanism of action of proteasome inhibitors and deacetylase inhibitors and the biological basis of synergy in multiple myeloma. Mol Cancer Ther. (2011) 10:2034–42. 10.1158/1535-7163.MCT-11-0433 22072815 PMC5527560

[23] Lichter DI Danaee H Pickard MD Tayber O Sintchak M Shi H Sequence analysis of β-subunit genes of the 20S proteasome in patients with relapsed multiple myeloma treated with bortezomib or dexamethasone. Blood. (2012) 120:4513–6. 10.1182/blood-2012-05-426924 23018640 PMC3757460

[24] Bennett EJ Bence NF Jayakumar R Kopito RR. Global impairment of the ubiquitin-proteasome system by nuclear or cytoplasmic protein aggregates precedes inclusion body formation. Mol Cell. (2005) 17:351–65. 10.1016/j.molcel.2004.12.021 15694337

[25] Santo L Hideshima T Kung AL Tseng JC Tamang D Yang M. Preclinical activity, pharmacodynamic, and pharmacokinetic properties of a selective HDAC6 inhibitor, ACY-1215, in combination with bortezomib in multiple myeloma. Blood. (2012) 119:2579–89. 10.1182/blood-2011-10-387365 22262760 PMC3337713

[26] Cosenza M Pozzi S. The Therapeutic Strategy of HDAC6 Inhibitors in Lymphoproliferative Disease. Int J Mol Sci. (2018) 19: E2337. 10.3390/ijms19082337 30096875 PMC6121661

[27] Lee HY Fan SJ Huang FI Chao HY Hsu KC Lin TE. 5-Aroylindoles act as selective histone deacetylase 6 inhibitors ameliorating Alzheimer's disease phenotypes. J Med Chem. (2018) 61:7087–102. 10.1021/acs.jmedchem.8b00151 30028616

[28] Chen CC Chow MP Huang WC Lin YC Chang YJ. Flavonoids inhibit tumor necrosis factor-alpha-induced up-regulation of intercellular adhesion molecule-1 (ICAM-1) in respiratory epithelial cells through activator protein-1 and nuclear factor-kappaB: structure-activity relationships. Mol Pharmacol. (2004) 66:683–93. 10.1124/mol.66.3 15322261

[29] Vogl DT Raje N Jagannath S Richardson P Hari P Orlowski R Ricolinostat, the first selective histone deacetylase 6 inhibitor, in combination with bortezomib and dexamethasone for relapsed or refractory multiple myeloma. Clin Cancer Res. (2017) 23:3307–15. 10.1158/1078-0432.CCR-16-2526 28053023 PMC5496796

[30] Mishima Y Santo L Eda H Cirstea D Nemani N Yee AJ. Ricolinostat (ACY-1215) induced inhibition of aggresome formation accelerates carfilzomib-induced multiple myeloma cell death. Br J Haematol. (2015) 169:423–34. 10.1111/bjh.13315 25709080

[31] Dankbar B Padró T Leo R Feldmann B Kropff M Mesters RM. Vascular endothelial growth factor and interleukin-6 in paracrine tumor-stromal cell interactions in multiple myeloma. Blood. (2000) 95:2630–6. 10753844

[32] Podar K Tai YT Davies FE Lentzsch S Sattler M Hideshima T. Vascular endothelial growth factor triggers signaling cascades mediating multiple myeloma cell growth and migration. Blood. (2001) 98:428–35. 10.1182/blood.V98.2.428 11435313

[33] Lin HY Chen CS Lin SP Weng JR Chen CS. Targeting histone deacetylase in cancer therapy. Med Res Rev. (2006) 26:397–413. 10.1002/med.20056 16450343

[34] Mercurio C Minucci S Pelicci PG. Histone deacetylases and epigenetic therapies of hematological malignancies. Pharmacol Res. (2010) 62:18–34. 10.1016/j.phrs.2010.02.010 20219679

[35] Sanchez E Shen J Steinberg J Li M Wang C Bonavida B. The histone deacetylase inhibitor LBH589 enhances the anti-myeloma effects of chemotherapy *in vitro* and *in vivo*. Leuk Res. (2011) 35:373–9. 10.1016/j.leukres.2010.06.026 20650529

[36] Eckschlager T Plch J Stiborova M Hrabeta J. Histone deacetylase inhibitors as anticancer drugs. Int J Mol Sci. (2017) 18:E1414. 10.3390/ijms18071414 28671573 PMC5535906

[37] Badros A Burger AM Philip S Niesvizky R Kolla SS Goloubeva O. Phase I study of vorinostat in combination with bortezomib for relapsed and refractory multiple myeloma. Clin Cancer Res. (2009) 15:5250–7. 10.1158/1078-0432.CCR-08-2850 19671864 PMC2758911

[38] Boyault C Sadoul K Pabion M Khochbin S. HDAC6, at the crossroads between cytoskeleton and cell signaling by acetylation and ubiquitination. Oncogene. (2007) 26:5468–76. 10.1038/sj.onc.1210614 17694087

[39] Kawaguchi Y Kovacs JJ McLaurin A Vance JM Ito A Yao TP. The deacetylase HDAC6 regulates aggresome formation and cell viability in response to misfolded protein stress. Cell. (2003) 115:727–38. 10.1016/S0092-8674(03)00939-5 14675537

[40] Bett JS. Proteostasis regulation by the ubiquitin system. Essays Biochem. (2016) 60:143–51. 10.1042/EBC20160001 27744330

[41] Hideshima T Bradner JE Wong J Chauhan D Richardson P Schreiber SL. Small-molecule inhibition of proteasome and aggresome function induces synergistic antitumor activity in multiple myeloma. Proc Natl Acad Sci USA. (2005) 102:8567–72. 10.1073/pnas.0503221102 15937109 PMC1150844

[42] Amengual JE Johannet P Lombardo M Zullo K Hoehn D Bhagat G. Dual targeting of protein degradation pathways with the selective HDAC6 inhibitor ACY-1215 and bortezomib is synergistic in lymphoma. Clin Cancer Res. (2015) 21:4663–75. 10.1158/1078-0432.CCR-14-3068 26116270 PMC4609274

[43] Imai Y Ohta E Takeda S Sunamura S Ishibashi M Tamura H. Histone deacetylase inhibitor panobinostat induces calcineurin degradation in multiple myeloma. JCI Insight. (2016) 1:e85061. 10.1172/jci.insight.85061 27699258 PMC5033869

[44] Wang J Hendrix A Hernot S Lemaire M De Bruyne E Van Valckenborgh E. Bone marrow stromal cell–derived exosomes as communicators in drug resistance in multiple myeloma cells. Blood. (2014) 124:555–66. 10.1182/blood-2014-03-562439 24928860

[45] Ding N Ping L Feng L Zheng X Song Y Zhu J. Histone deacetylase 6 activity is critical for the metastasis of Burkitt's lymphoma cells. Cancer Cell Int. (2014) 14:139. 10.1186/s12935-014-0139-z 25546298 PMC4276069

[46] Mahlknecht U Schönbein C. Histone deacetylase inhibitor treatment downregulates VLA-4 adhesion in hematopoietic stem cells and acute myeloid leukemia blast cells. Hematologica. (2008) 93:443–6. 10.3324/haematol.11796 18268283

[47] Noborio-Hatano K Kikuchi J Takatoku M Shimizu R Wada T Ueda M. Bortezomib overcomes cell adhesion-mediated drug resistance through downregulation of VLA-4 expression in multiple myeloma. Oncogene. (2009) 28:231–42. 10.1038/onc.2008.385 18850009

[48] Huang YC Huang FI Mehndiratta S Lai SC Liou JP Yang CR. Anticancer activity of MPT0G157, a derivative of indolylbenzenesulfonamide, inhibits tumor growth and angiogenesis. Oncotarget. (2015) 6:18590–601. 10.18632/oncotarget.4068 26087180 PMC4621912

