# Supplementary figures and images for: MPT0G413, A Novel HDAC6-Selective Inhibitor, and Bortezomib Synergistically Exert Anti-tumor Activity in Multiple Myeloma Cells
Source: Front Oncol. 2019 Apr 9;9:249. doi: 10.3389/fonc.2019.00249 (PMC6465934; doi:10.3389/fonc.2019.00249)

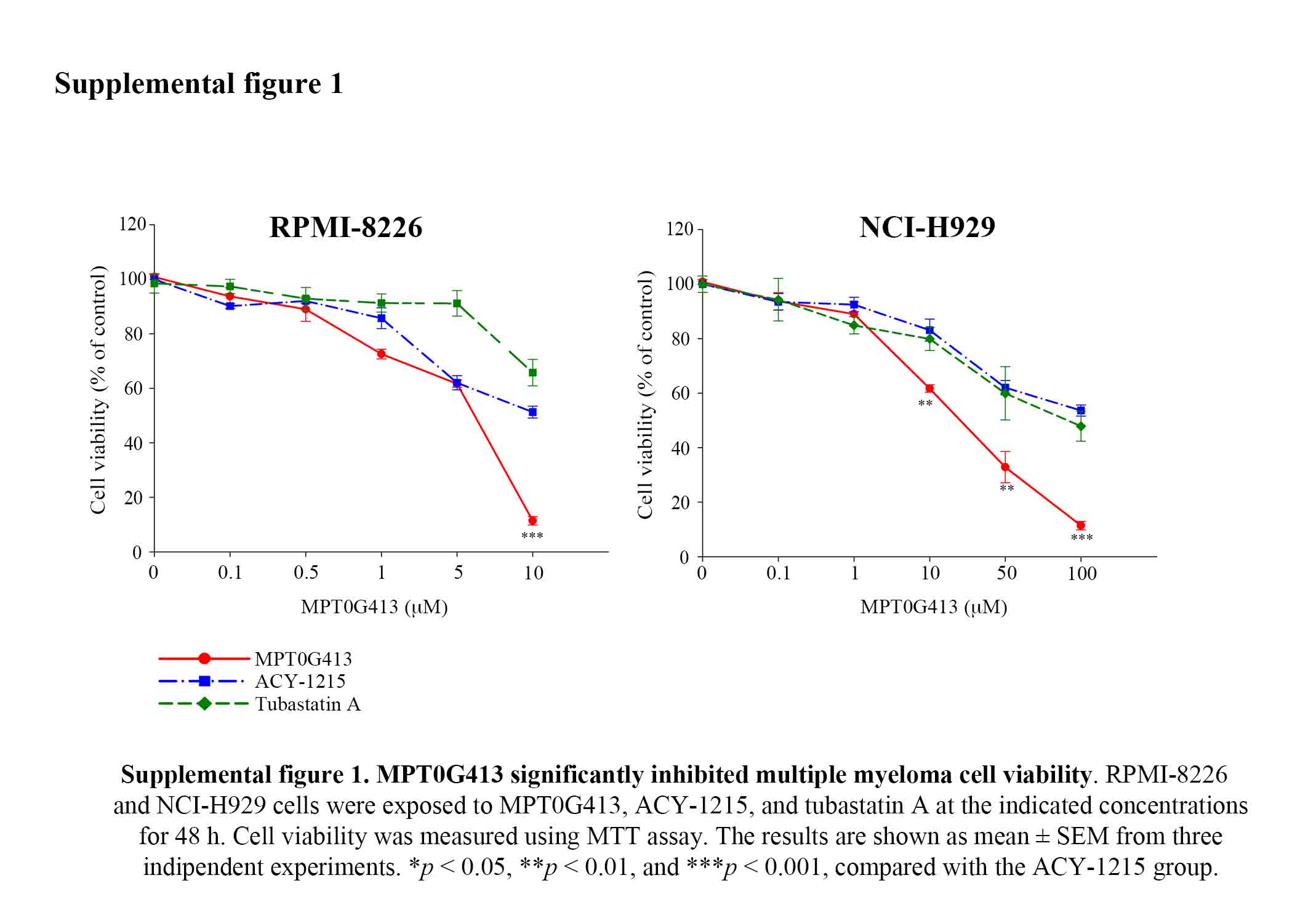

Supplement: Supplementary file 1 [file Image_1.JPEG]

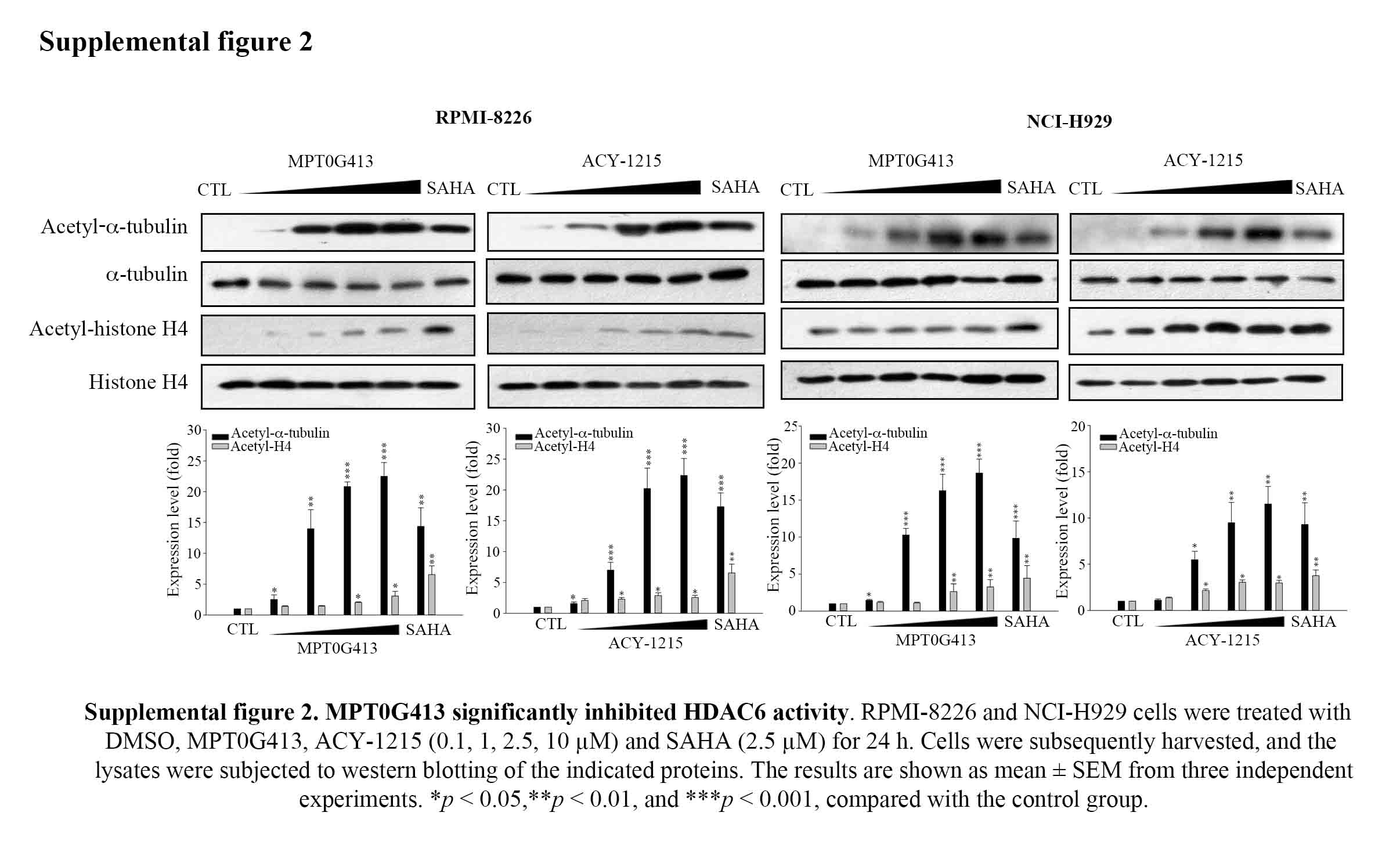

Supplement: Supplementary file 2 [file Image_2.JPEG]

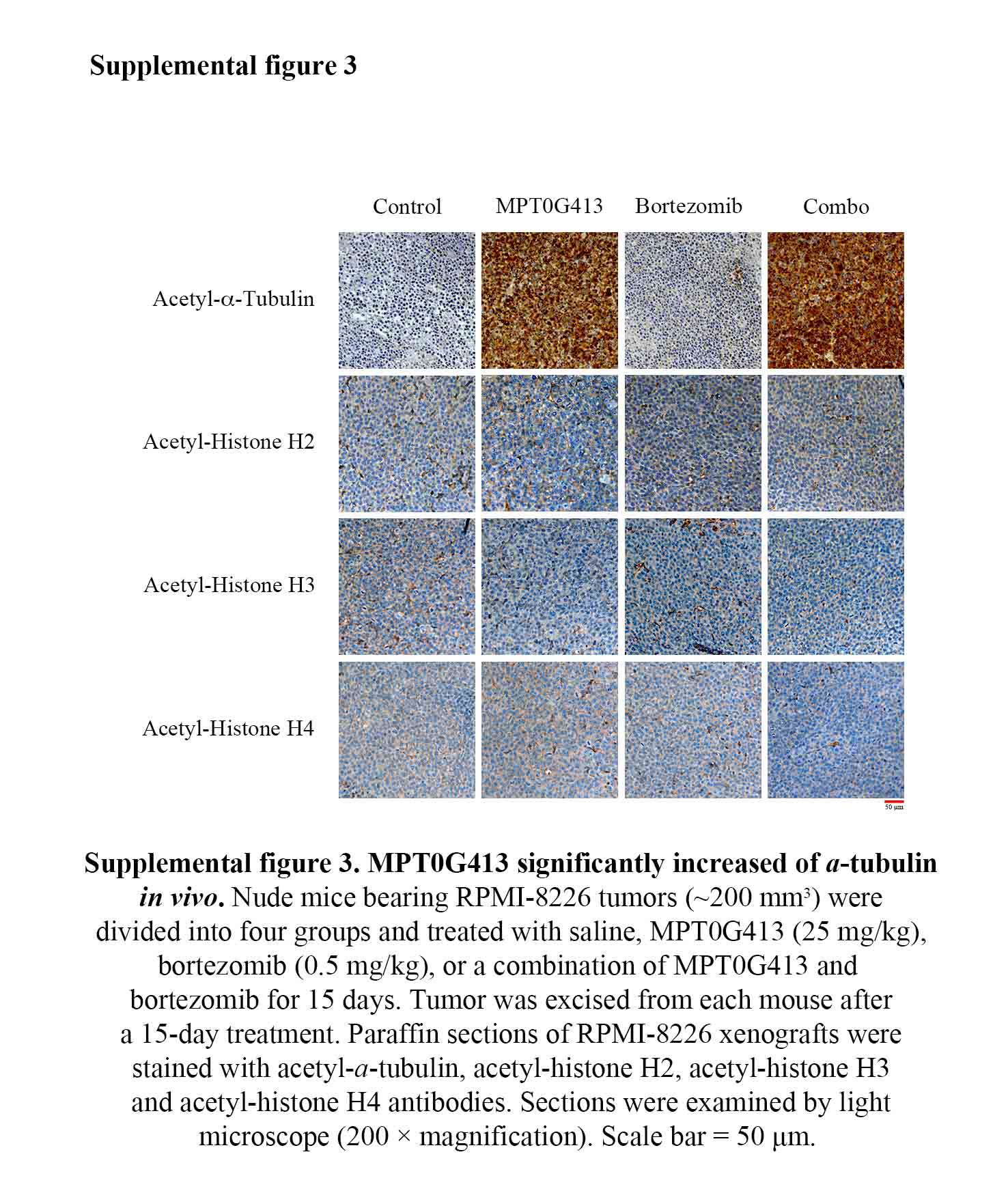

Supplement: Supplementary file 3 [file Image_3.JPEG]
